# Natural course of pineal cysts—a radiographic study

**DOI:** 10.1186/s41016-018-0142-7

**Published:** 2018-12-11

**Authors:** Martin Majovsky, Vladimir Benes

**Affiliations:** Department of Neurosurgery and Neurooncology, First Faculty of Medicine, Charles University and Military University Hospital, U Vojenské nemocnice 1200, Prague 6, Czech Republic

**Keywords:** Pineal cyst, Natural history, Magnetic resonance imaging, Neurosurgery

## Abstract

**Background:**

Pineal cysts (PCs) are a benign lesion of the pineal gland that have been known to the medical community for a long time. With a prevalence rate of approximately 1% in the general population, PC is often a reason for medical counseling. The natural course of PC morphology has not been well described. In this study, we present a longitudinal magnetic resonance imaging (MRI) study of patients with PCs, with special focus on those who showed an increase or decrease in PC size.

**Methods:**

We enrolled all patients with a PC who were referred to our department between January 2000 and January 2018. Each patient underwent a clinical examination, and the patient’s age, sex, and presenting signs and symptoms were noted. MRI was performed during periodic examinations, and a clinical and radiological course was reassessed.

**Results:**

In total, 133 patients (99 women, 34 men) were enrolled. The mean maximum diameter was 12.7 ± 5.2 mm (range 7–35 mm). PCs increased in size during the follow-up in seven patients (5.3%) and decreased in size in 10 (7.5%). The remaining cysts (*n* = 116, 87.2%) were stable over the follow-up period. Analyzing patients according to cyst size change, we found a significant difference in the mean age between the PC progression group and PC regression group (*p* = 0.01). The mean size of the PCs at the time of diagnosis did not differ significantly between the two groups (*p* = 0.81). We diagnosed two cases of pineal apoplexy.

**Conclusion:**

We found that PCs are a dynamic structure that may change in size during the patient’s lifetime. Patients with an increase in PC size were significantly younger than patients with a decrease in size. Therefore, PC growth in the first, second, and third decennium is normal and does not justify medical intervention. Surgery is indicated in cases of hydrocephalus and Parinaud’s syndrome or in atypical cysts when neoplasia is suspected. The size of a PC does not predict PC behavior in terms of a future increase or decrease in size.

## Background

Pineal cysts (PCs) are a benign lesion of the pineal gland that have been known to the medical community for a long time. The founder of modern pathology, Rudolf Virchow, described this entity as *hydrops cysticus glandulae pinealis* in 1865 [1], and Campbell provided the first detailed description of its histological structure in 1899 [2]. The first surgical procedure on a PC was published by Pussep et al. in 1914 in a boy with hemorrhagic apoplexy of the gland [3].

The pathogenesis of the PC remains unknown despite that numerous hypotheses have been formulated over the years. One possible mechanism to account for PC development is mild brain hypoxia in the perinatal period [4]. Ozmen et al. reported a higher prevalence of PCs in patients with cerebral palsy and periventricular leukomalacia [5]. Some studies suggest that pineal necrosis (apoplexy) may lead to the subsequent creation of a glial scar in the form of a cyst [6, 7]. Other studies have proposed that PCs may originate from sequestration of the pineal recess of the third ventricle [8, 9].

With a prevalence rate of approximately 1% in the general population, PC is often a reason for medical counseling. The natural course of PC morphology has not been well described: PC might increase in size, decrease in size, and may even form de novo. Knowledge of the natural course of PCs is very important, especially in a situation when PC growth is detected during follow-up. In this scenario, several questions typically arise: Is the diagnosis of PC correct? Is there suspicion of neoplasia? Is PC growth the cause of the presenting symptoms? Is surgery indicated?

In this study, we present a longitudinal magnetic resonance imaging (MRI) study of patients with PCs, with special focus on those who showed an increase or decrease in PC size.

## Methods

### Patients

We enrolled all patients with a PC who were referred to our department between January 2000 and January 2018. Each patient underwent a clinical examination, and the patient’s age, sex, and presenting signs and symptoms were noted. MRI was performed during periodic examinations, and a clinical and radiological course was reassessed. Exclusion criteria were known structural brain disease, previous craniotomy, and insufficient relevant data.

### Radiographic evaluation

PC was defined as a smooth-walled cystic lesion in the pineal region and rim enhancement after gadolinium not thicker than 2 mm on MRI. The size of the PC was measured as the largest diameter on a sagittal T2-weighted image. Attention was given to the size of the change in the PC, i.e., size increase or size decrease. The change in size was defined as a difference of at least 2 mm in the largest diameter at the time of follow-up. A lower cutoff value for size was 7 mm. A multilobular structure of the PC was not considered a reason for exclusion. Serial high-field MRI (3T) scans with gadolinium enhancement were acquired every 1 to 3 years during the follow-up.

### Statistical analysis

Comparison of continuous variables was done using Student’s *t* test (two-tailed) for unequal variances. Statistical significance was set at *p* = 0.05. Statistical analysis was performed using STATISTICA version 12.0 software (Stat-Soft, Tulsa, OK, USA).

## Results

**Fig. 1:**
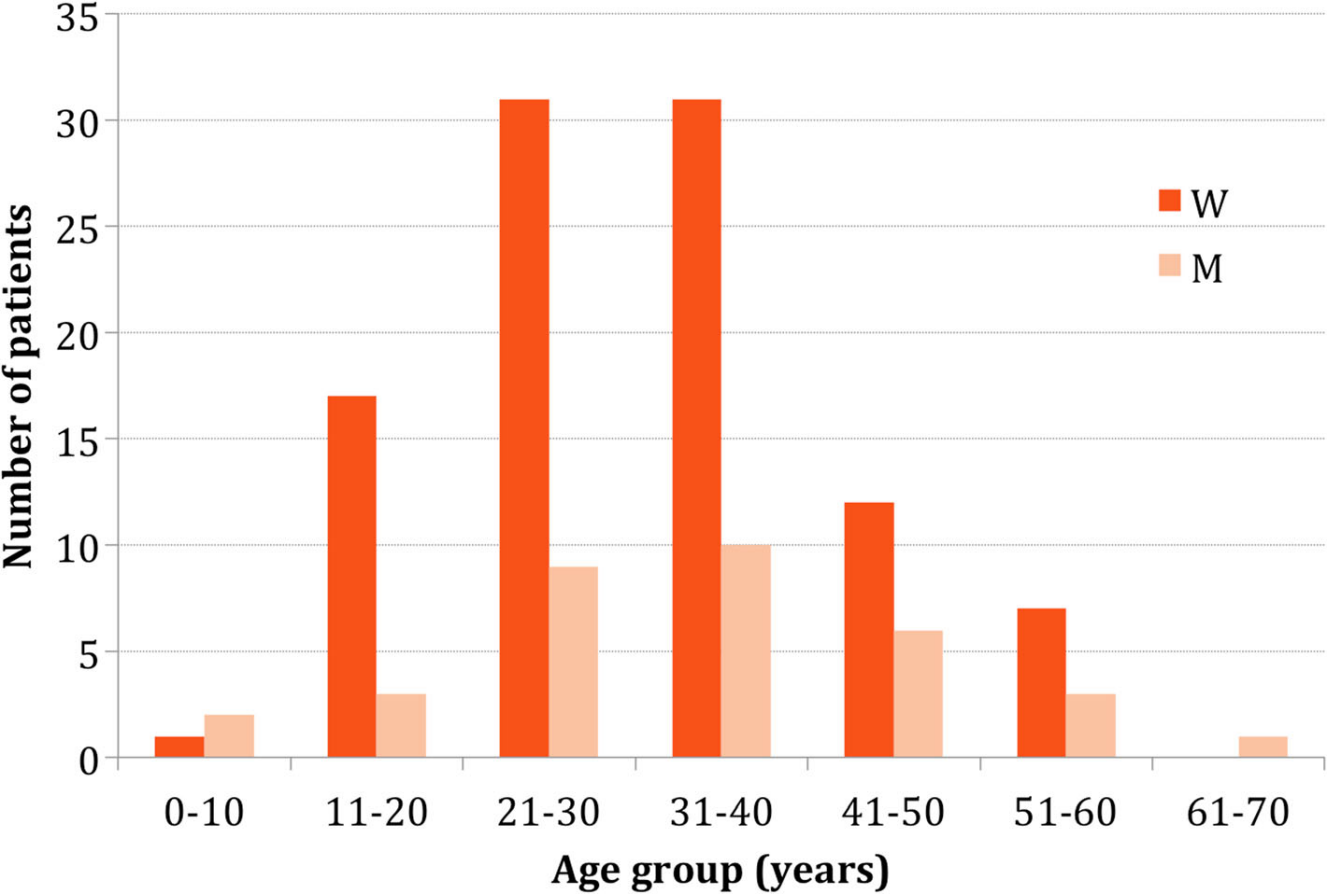
Age and sex distribution of patients with pineal cysts (W women, M men)

In total, 133 patients (99 women, 34 men) were enrolled. The mean age at the time of diagnosis was 31.1 ± 12.2 years (range 7–62 years) (Fig. 1).

**Fig. 2:**
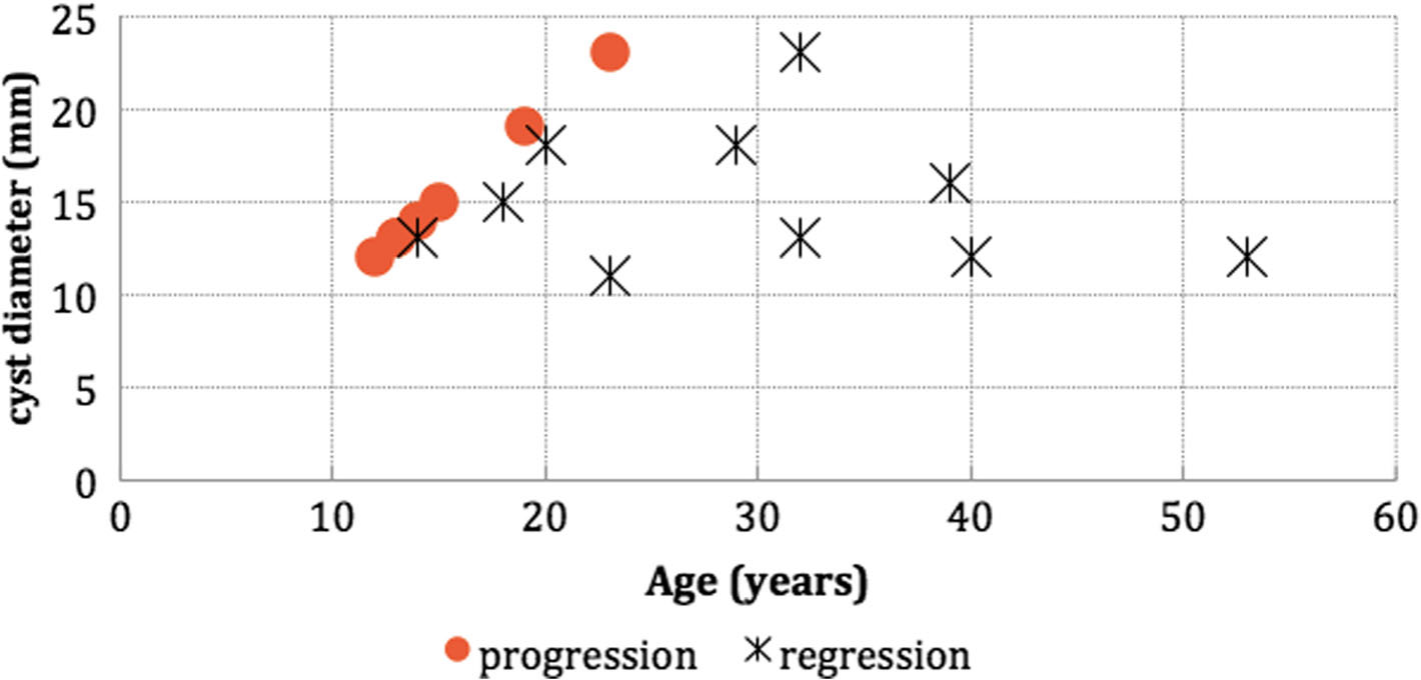
Scatter chart showing patients with pineal cyst progression and regression by age and cyst diameter

The mean maximum diameter was 12.7 ± 5.2 mm (range 7–35 mm). PCs increased in size during the follow-up in seven patients (5.3%) and decreased in size in 10 (7.5%) (Fig. 2). The remaining cysts (*n* = 116, 87.2%) were stable over the follow-up period.

Analyzing patients according to cyst size change, we found a significant difference in the mean age between the PC progression group and PC regression group (*p* = 0.01). The mean size of the PCs at the time of diagnosis did not differ significantly between the two groups (*p* = 0.81).

**Fig. 3:**
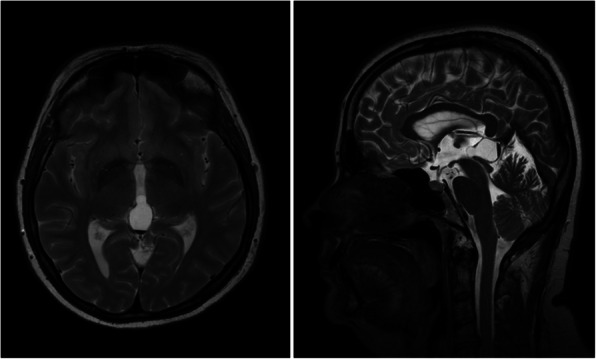
Patient with pineal apoplexy. Left: axial T2-weighted image showing large pineal cyst occupying posterior third ventricle and pineal region. Note the fluid-fluid level showing blood sediment. Right: sagittal T2-weighted image showing the same finding

We diagnosed two cases of pineal apoplexy based on newly formed fluid-fluid level on a MRI scan (Fig. 3). One female patient, aged 41 years, known to have a PC experienced sudden onset of headache and nausea. Physical and laboratory examinations showed no (detectable) abnormalities, however. A CT scan and subsequent MRI indicated a newly formed blood level in the PC. Although symptoms subsided within 1 week, the patient has since suffered from frequent headaches. The second patient, a 31-year-old pregnant woman with known PC, experienced one episode of expressive aphasia, phosphenes, and limb paresthesia in the 27th week of gestational age. She was on low-dose enoxaparin (0.2 ml per day) because of elevated D-dimer. An MRI scan showed signs of bleeding into the PC. Uncomplicated vaginal delivery occurred in the 36th week of pregnancy. The patient is now symptom free, and similar episodes have not recurred. No change in PC size was observed in either patient after apoplexy.

## Discussion

We diagnosed most of the PCs in patients in their third and fourth decennium (i.e., 21–40 years). Our results are obviously biased given that we normally do not treat pediatric patients in our department. The largest study on the natural course of PCs reviewed 63,933 consecutive patients who underwent an MRI examination [10]. The authors found a peak prevalence of PCs (3.7%) in the age group 6–12 years, followed by a steady decline in older age groups, reaching 0.1% in the oldest age group (81–90 years) [10, 11].

Nevins et al. reviewed 42,099 consecutive patients aged ≥ 16 years who underwent a MRI examination [12]. The authors reported an overall prevalence of 0.7%, peaking in the age group 16–19 years. Sawamura et al. reviewed the MRI scans of 6023 patients and found PCs in 1.3% of this large patient group [13]. No PCs were found in patients < 10 years old, with the peak incidence occurring in the age group 21–30 years. Based on these data, PC growth in the first, second, and third decade of life appears normal and hence does not justify surgical intervention. Surgical treatment is reserved for patients with obstructive hydrocephalus and Parinaud’s syndrome. In an acute setting, external ventricular drainage could be placed as an emergency process to allow temporary drainage of cerebrospinal fluid (CSF), relieving elevated intracranial pressure [14]. Restoration of a physiological circulation of the CSF by PC removal is a goal, and the outcome for these patients is usually excellent. The surgical treatment of patients with PCs presenting with non-specific symptoms is highly controversial within the neurosurgical community [15]. Surgery might also be considered in atypical cases in which the histological diagnosis is uncertain, especially in cases of rapid growth, multilobular PCs, or heterogenous contrast enhancement, which may arouse suspicion of a tumor. Several cases have been described in the literature in which pineal tumors were misdiagnosed as PCs [16, 17].

The female to male ratio in the present study was 2.91:1. Al-Holou et al. reported a lower ratio of 1.95:1 [10], Nevins 1.73:1 [12], and Barboriak 1.46:1 [18]. The larger number of female to male patients in our study might be due to tension headache and migraines that are more common in women. Some authors speculate that the female preponderance of PCs is due to the influence of hormones [19, 20].

In our series, PCs increased in size during the follow-up in seven patients (5.3%) and decreased in size in 10 patients (7.5%) while the remaining PCs (87.2%) remained stable. Nevins et al. reported a MRI series of 181 patients with PCs who underwent more than one examination [12]. The median follow-up in this study was only 6 months. The authors noted an increase in size in 3.9% of the patients with a median age of 44 years and a decrease in size in 2.2% of the patients with a median age 30 years. Al-Holou et al. reported a MRI series in 151 patients with PCs aged > 19 years who had undergone more than one examination [10]. The median follow-up in this study was 3.4 years. The authors observed an increase in size in 2.6% of the patients and a decrease in size in 15%. Patients with an increase in cyst size had lower age at the time of diagnosis than patients with decrease in cyst size, but this difference was not statistically significant. Al-Holou et al. reported another MRI series of 106 patients with PCs < 26 years of age with a mean follow-up of 3 years [11]. The authors reported an increase in size in 5.7% of the patients and a decrease in size in 0.9%.

Two cases of pineal apoplexy, a rare apoplectic event, were noted in our series, both presenting with rather mild symptoms. A typical clinical presentation described in the literature is the sudden onset or acute worsening of such symptoms as headache, gaze paresis, nausea/vomiting, syncope, and ataxia [6, 21]. In certain cases, acute bleeding into pineal gland may cause a rapid increase in size and acute hydrocephalus, which requires immediate surgical intervention. Cases of sudden death due to apoplexy have been described [22]. Some authors suggest that pineal apoplexy may be a causative factor in the formation of a PC [6, 7]. We did not find a change in PC size after apoplexy in our two patients.

## Conclusions

We found that PCs are a dynamic structure that may change in size during the patient’s lifetime. Age was the risk factor for growth, while initial size was not. Surgery is indicated in cases of hydrocephalus and Parinaud’s syndrome or in atypical cysts when neoplasia is suspected. The size of a PC does not predict PC behavior in terms of a future increase or decrease in size.

## Abbreviations

CSF: Cerebrospinal fluid
CT: Computed tomography
MRI: Magnetic resonance imaging
PC: Pineal cyst

## Authors’ information

MM is a senior resident in the Department of Neurosurgery and Neurooncology of First Faculty of Medicine, Charles University and Military University Hospital, in Prague, Czech Republic. He received his PhD degree in Neuroscience on the thesis “Pineal lesions: clinical presentation, hormone secretion, sleep quality and effect of surgical treatment”.
